# Advances in heterometallic ring-opening (co)polymerisation catalysis

**DOI:** 10.1038/s41467-021-23192-y

**Published:** 2021-05-31

**Authors:** Weronika Gruszka, Jennifer A. Garden

**Affiliations:** EaStCHEM School of Chemistry, University of Edinburgh, Edinburgh, UK

**Keywords:** Homogeneous catalysis, Polymer synthesis

## Abstract

Truly sustainable plastics require renewable feedstocks coupled with efficient production and end-of-life degradation/recycling processes. Some of the most useful degradable materials are aliphatic polyesters, polycarbonates and polyamides, which are often prepared via ring-opening (co)polymerisation (RO(CO)P) using an organometallic catalyst. While there has been extensive research into ligand development, heterometallic cooperativity offers an equally promising yet underexplored strategy to improve catalyst performance, as heterometallic catalysts often exhibit significant activity and selectivity enhancements compared to their homometallic counterparts. This review describes advances in heterometallic RO(CO)P catalyst design, highlighting the overarching structure-activity trends and reactivity patterns to inform future catalyst design.

## Abstract (web summary)

Improving catalyst performance for ring-opening (co)polymerisation (RO(CO)P) reactions by using heterometallic cooperativity is a promising yet underexplored strategy. Here the authors review advances in heterometallic RO(CO)P catalyst design, highlighting the overarching structure-activity trends and reactivity patterns to inform future catalyst design.

## Introduction

In nature, heterometallic enzymes enable a variety of efficient catalytic transformations^1,2^, where the relative proximity of the substrates is a key feature and is controlled by the metals. Inspired by this, chemists have developed heterometallic complexes where two different metals held within the same molecular environment can work together to create a “cooperative” effect. Cooperative heterometallic complexes are often “greater than the sum of their parts”, outperforming the homometallic counterparts in terms of activity and selectivity, or enabling chemical transformations that are otherwise inaccessible^3–7^. This concept has been exploited across multiple reactions, including metal-halogen exchange^8^, C–H activation^9^ and asymmetric catalysis^10^. Heterometallic cooperativity has vast potential to improve catalyst activity, and the “Pairodic Table of Element Pairs” emphasises the extensive number of heterometallic combinations available^6^, each leading to unique reactivities^3,6^. However, this approach remains underexplored, with most organometallic catalyst design focused on ligand modification. Understanding the origins of heterometallic cooperativity is crucial to improve catalyst design and harness the full potential of this strategy.

**Fig. 1:**
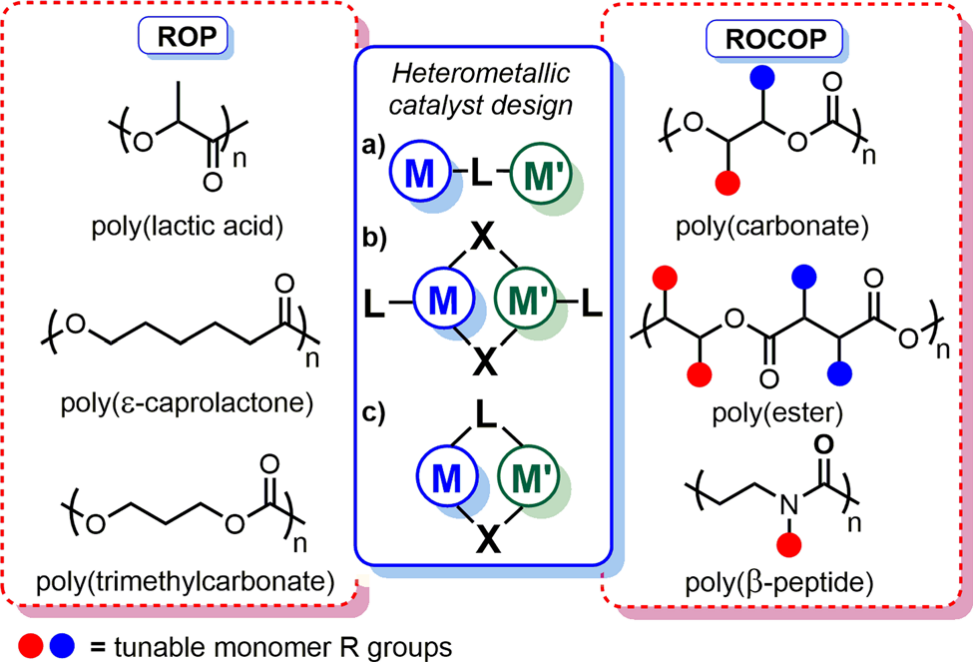
Heterometallic structural motifs and polymerisation products highlighted in this review. L = ligand, X = bridging or exchangeable ligand (e.g. nitrogen, oxygen, halogen).

Heterometallic complexes can operate via multisite interactions with each metal catalysing different reaction steps^3^, or with one metal acting as the primary catalytic site and the other metal(s) modulating its reactivity. This is often dictated by the structure. When bound to different heteroatoms in the ligand (Fig. 1a), the metal-metal’ distances (M–M′, where M ≠ M′) are often dependent on the ligand flexibility and multisite interactions may be favoured. Electronic communication can arise when two metals are connected through a heteroatom in dimeric and dinucleating systems (Fig. 1b, c), resulting in short M–M′ distances^7^. This electronic modulation can also occur in “ate” complexes (e.g. lithium magnesiate LiMgR_3_ or lithium zincate LiZnR_3_), where a hard metal M is paired with a softer, more carbophilic metal M′ (e.g. Li^+^ with MgR_3_^–^/ZnR_3_^–^)^4,6,11^. This anionic “ate” activation can increase the nucleophilicity and/or Brønsted basicity of the M′-*R* group, while concomitantly enhancing the Lewis acidity of the M^+^ cation. Heterometallic complexes may also feature direct polar M–M′ bonds^12^, providing access to unique reactivities^3^.

Whilst heterometallic cooperativity has been well-studied in a range of organic transformations, heterometallic catalysis is still gathering momentum in polymerisation processes^7,13^. With increased demand for sustainable plastics, research into ring-opening polymerisation (ROP) and ring-opening copolymerisation (ROCOP) to produce useful and degradable polymers has grown rapidly. Two of the most promising current strategies^14^ are the production of poly(lactic acid) (PLA) via ROP of bioderived lactide (LA)^15^, and carbon dioxide (CO_2_)/epoxide ROCOP to form polycarbonates and polyurethanes^16^. The material properties are dictated by the polymer microstructure (e.g. chain length, dispersity and tacticity) and organometallic RO(CO)P catalysts have generally outperformed organocatalysts and enzymes in combining activity with polymerisation control^17^.

Heterometallic catalysts have the potential to revolutionise RO(CO)P by providing multiple and inequivalent catalytic sites for monomer activation and nucleophilic attack, which are key steps during initiation and propagation. The catalyst performance is controlled by the metals, ligand architecture and the polymerisation conditions. Importantly, not all metal combinations are cooperative. While isolated studies have been reported, this review identifies key structural motifs and overarching heterometallic activity trends across cyclic ester/carbonate ROP, epoxide and CO_2_/cyclic anhydride ROCOP and aziridine/carbon monoxide (CO) ROCOP (Fig. 1), in order to guide future heterometallic catalyst development.

## Ring-opening polymerisation

Cyclic ester ROP is an efficient route towards degradable aliphatic polyesters with engineering, packaging^18^ and biomedical applications^17,19,20^. Metal-catalysed ROP typically proceeds through a coordination-insertion mechanism with the catalysts comprising a Lewis acidic metal and a nucleophile (e.g. alkoxide/amide) supported by sterically-hindered ligands^17,20,21^. While many homometallic ROP catalysts have been reported^21–25^, some multimetallic complexes (e.g. bis-Al, Hf, In, Mg, Ti, Y, Zn and Zr) have shown significant activity and selectivity enhancements^24–28^, and some of which have been proposed to operate via a chain-shuttling mechanism^29,30^. There is a substantial opportunity to further improve catalyst performance through heterometallic cooperativity, and progress has already been made with heterocombinations from across the Periodic Table. While detailed mechanistic studies have not yet been reported for heterometallic complexes, experimental observations indicate that the larger and more electropositive metal acts as the monomer coordination site, and the more Lewis acidic metal acts as the source of the M-alkoxide nucleophile^31^. Complexes where alkali metals (K/Li/Na) are combined with divalent (Mg/Zn)^31–35^, trivalent (Al/In/Y)^36–40^ or tetravalent (Ge/Sn) elements have been most prevalent in ROP^41^. Combining non-toxic and earth abundant metals such as Al, Mg and Zn with alkali metals is particularly appealing from both economic and environmental perspectives^42^.

### Alkali metal/divalent metal heterocombinations

**Fig. 2:**
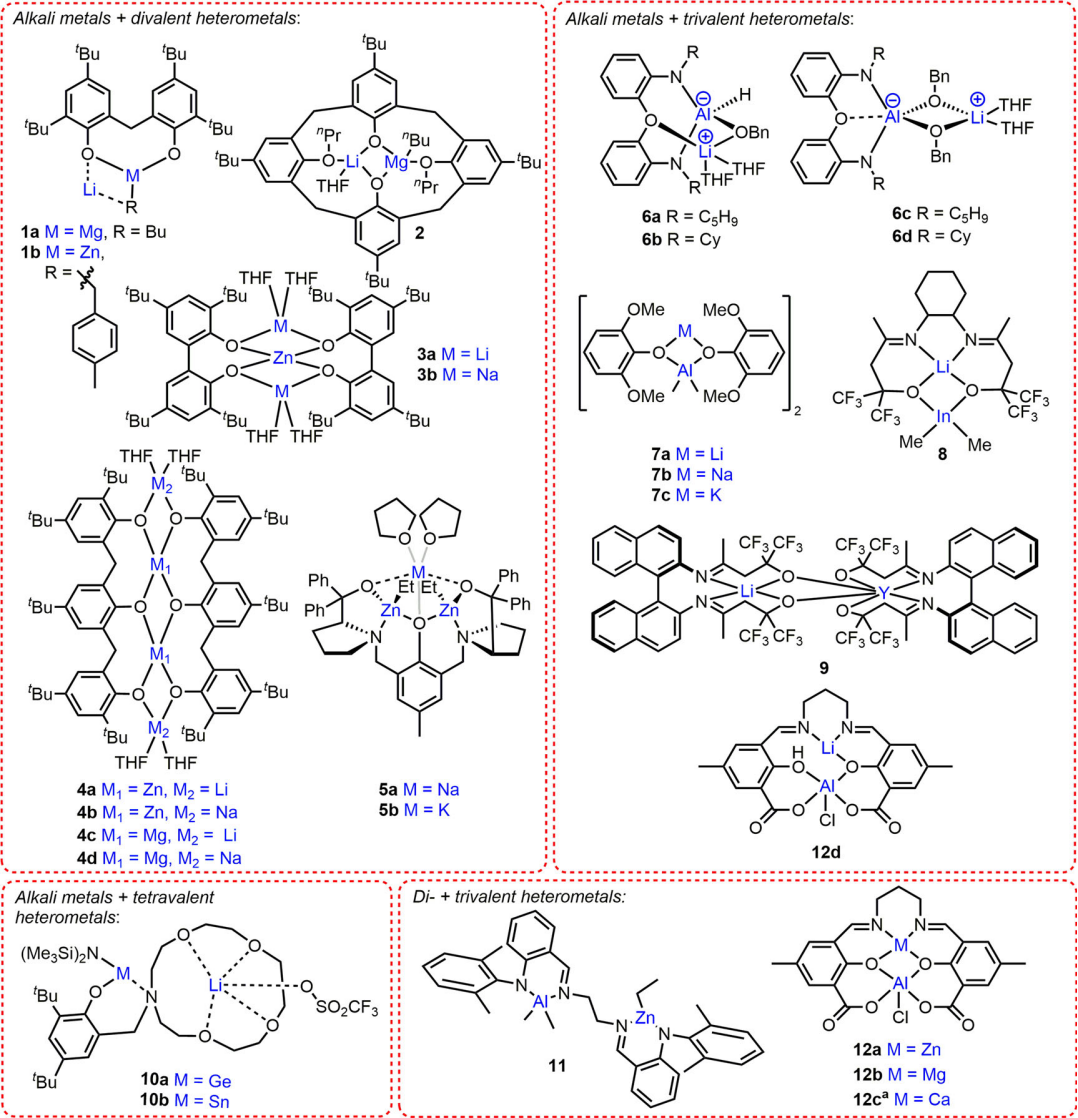
Main group heterometallic catalysts for cyclic ester ROP. Structural representations of heterometallic complexes reported for cyclic ester ROP, where alkali metals are combined with di-, tri- and tetravalent heterometals and trivalent Al is combined with divalent metals. ^a^In **12c**, one Ca bridges two (salen)AlCl complexes with each ligand retaining one phenolic O*H* group.

In situ generated Li/Zn and Li/Mg complexes **1a-b** (Fig. 2) both displayed good activities at ambient temperature, converting 62 equiv. and 88 equiv. *rac*-LA in 15 min, respectively (*Đ* = 1.18 (**1a**), 1.31 (**1b**)), with 1 equiv. neopentyl alcohol^32^. Interestingly, **1a** exhibited higher activity and stereocontrol (*P*_s_ = 0.87–0.88) in 5:1 toluene:THF than in toluene (activity) or THF (stereocontrol). Solvent choice can significantly influence heterometallic solution-state structures, and the reduced stereocontrol in THF may arise from the in situ formation of less sterically hindered solvent separated Li^+^MR_3_^–^ (M = Mg, Zn) ion pairs^43^. Solution-state structural analysis is therefore crucial for uncovering differences between solvent separated and contact ion pairs, and understanding how these changes influence catalyst activity within ROP^5^.

Heterometallic Li/Mg complex **2** (Fig. 2, Li–Mg distance = 2.67 Å), converted 55 equiv. *rac*-LA in 1 h with 1 equiv. MeOH in DCM at 20 °C (*Đ* = 1.22)^33^. The experimental *M*_n_ values were almost double those calculated, which was attributed to slow initiation via in situ generated Mg–Cl species (by reaction of Mg-^*n*^Bu with DCM). The mono-Mg complex displayed similar control (*Đ* = 1.12), however this catalyst required 2 h to convert 55 equiv. *rac*-LA under otherwise identical conditions.

Lithium and sodium zincates **3a-b** (Fig. 2) exhibited similar activity, polymerising 182 equiv. and 190 equiv. l-LA, respectively (toluene, 48 h, 90 °C)^34^, with relatively good control (*Đ* = 1.42 and 1.26, respectively) and generating OH-terminated PLA chains. **3b** was also active and controlled under non-anhydrous conditions, converting 87 equiv. l-LA in 48 h at 90 °C (*Đ* = 1.33). This was attributed to the partial dissociation of **3b** to form the mono-Na aryloxide complex in situ, with the latter shown to be more active in l-LA ROP (138 equiv. l-LA converted in 24 h, anhydrous conditions, *Đ* = 1.33), however the solution-state structures of **3a-b** were not investigated. **3a** generated only trace PLA under non-anhydrous conditions, which suggested that complete hydrolysis occurred. A trisphenol ligand was used to synthesise tetrametallic M_2_/Zn_2_ and M_2_/Mg_2_ (M = Li or Na) complexes **4a-d** (Fig. 2)^35^. The additional Zn centre in **4b** may enhance the activity vs. **3b** (238 equiv. l-LA converted in 36 h at 110 °C, *Đ* = 1.31)^34^, albeit direct comparison could not be made due to the different reaction conditions.

We recently reported Na/Zn_2_ and K/Zn_2_ complexes **5a-b** (Fig. 2) for LA, ε-caprolactone (ε-CL) and δ-valerolactone (δ-VL) ROP^31^, which outperformed the homometallic counterparts by combining high activities (Na/K) with good control (Zn_2_). In the presence of 2 equiv. benzyl alcohol (BnOH), **5b** (K/Zn_2_) converted 60 equiv. *rac*-LA in just 20 s (THF, room temperature, *Đ* = 1.40, *k*_obs_ = 1.7 × 10^−2^ s^−1^); to date, this is the fastest heterometallic catalyst system reported for LA ROP. NMR spectroscopy (including DOSY) and density-functional theory calculations suggested that **5a-b** retain their heterometallic structures in the solution-state. **5a-b** display improved activities in Lewis donor THF (vs. the analogous bis-Zn complex), and the fivefold activity increase in LA ROP upon switching Na (**5a**) for the larger K centre (**5b**) highlighted the key role of Na/K in LA coordination and activation. In both cases, incorporating Na/K also labilised the Zn-Et bonds (vs. the bis-Zn complex), as evidenced by NMR spectroscopy, accelerating the nucleophilic attack and LA ring-opening.

### Alkali metal/trivalent metal heterocombinations

Sterically-hindered mono-alkoxide Li/Al complexes **6a-b** (Fig. 2) were inactive, however the bis-alkoxide **6c-d** analogues polymerised LA at room temperature^36^, which is uncommon for Al-based catalysts^23,26^. **6c-d** were still relatively slow, converting 189 equiv. *rac*-LA in 16 h in DCM, albeit with good control (*Đ* = 1.03)^36^ compared to other Group 1 catalysts^23^. The enhanced activity of **6c-d** vs. Al-based catalysts was tentatively attributed to their in situ dissociation to [(RN-*o*-C_6_H_4_)_2_O)Al(OBn)] (R = C_5_H_9_ or Cy) and LiOBn. However, these monometallic counterparts were inactive under the conditions employed with **6c-d**, providing evidence for cooperative Li/Al operation in **6c-d**.

Preliminary results with M/Al complexes **7a-c** (M = Li, Na, K respectively, Fig. 2) indicated that **7a** and the analogous bis-Al complex were more active than **7b-c** in ROP^37^. **7a** and the bis-Al analogue converted 78 and 75 equiv. l-LA, respectively, whereas **7b-c** polymerised 48 and 20 equiv. l-LA, respectively (1 equiv. BnOH, 5 h, 125 °C, toluene). The higher activity of **7a** vs. **7b-c** was not specifically addressed but deviates from the activity trend commonly observed for Group 1 ROP catalysts (Li^+^ < Na^+^ < K^+^), where larger metals typically enhance monomer coordination thus polymerisation activity^44^. NMR analysis indicates the (L)Al-*Me*_2_ groups are more nucleophilic in **7a-c** than in the bis-Al species. The activity differences observed suggest a combination of multiple factors are important, including the metals, ligand and solution-state structures.

The Li/In complex **8** (Fig. 2) converted 98 equiv. *rac*-LA in 30 min with 1 equiv. isopropanol (^*i*^PrOH), and 96 equiv. *rac*-LA in 1 h without ^*i*^PrOH (toluene, 80 °C)^38^. Polymerisation control was poor in both cases, albeit slightly improved without ^*i*^PrOH (*Đ* = 2.16 vs. 2.56 with O^*i*^Pr). The reduced control with **8**/^*i*^PrOH may arise from competitive “activated monomer” and coordination-insertion mechanisms, as evidenced by both O^*i*^Pr and Me PLA end groups. As the synthesis of [{ON^Cy^NO}In(Me)] proved challenging, the activity of **8** was compared to homometallic [{ON^Cy^NO}In(CH_2_SiMe_3_)], which was significantly slower converting 93 equiv. *rac*-LA in 15 h (no ^*i*^PrOH, toluene, 80 °C); the reduced activity was attributed to slower initiation by the less nucleophilic In-CH_2_SiMe_3_.

In comparison to **8**, the Li/Y complex **9** (Fig. 2) exhibited higher activity in ROP without an alcohol (BnOH), converting 225 equiv. *rac*-LA to form heterotactic PLA (*P*_s_ = 0.99) in 5 h at 30 °C in THF (vs. 68 equiv. in 7 h at 70 °C with 1 equiv. BnOH)^39^. DOSY NMR analysis confirmed the heterometallic structure of **9** in THF solvent. Initiation was proposed to occur via nucleophilic attack from the ligand of **9** based on SEC, ^1^H NMR spectroscopy and MALDI-ToF analysis.

### Alkali metal/tetravalent metal heterocombinations

The Li/Ge complex **10a** (Fig. 2) was almost twice as active as the mono-Ge analogue in l-LA ROP (57% vs. 35% PLA yield, respectively; 500 equiv. l-LA, 10 equiv. ^*i*^PrOH, 6 h, 100 °C), which may arise from Li^+^ enhancing l-LA coordination^41^. In contrast, the Li/Sn complex, **10b**, was slower than the homometallic mono-Sn analogue, which was attributed to the higher moisture- and air-sensitivity of the former and possible catalyst decomposition during ROP.

### Divalent/trivalent metal heterocombinations

The Al/Zn complex **11** (Fig. 2) was more active than the analogous mono-Al and bis-Al complexes in ε-CL ROP^45^, resulting in 95% PCL yield in 6 min at 70 °C (2:1:100 BnOH:catalyst:ε-CL)^46^. It was, however, slower than the bis-Zn analogue^45^, which produced 98% PCL in 1 min under the same conditions suggesting that Zn is more catalytically active than Al in **11**^46^. The higher activity of Zn was attributed to the lower bond dissociation energy of Zn-O (284 kJ mol^−1^) vs. Al-O (512 kJ mol^−1^); M-OR bond cleavage is a key step in ROP.

Our group reported Al/Zn and Mg/Al complexes **12a-b**^40^, which displayed good catalyst activities in *rac*-LA ROP, outperforming the mono-Al analogue by respective factors of two and 11 under identical conditions (**12a**, *k*_obs_ = 1.8 × 10^−3^ s^−1^; **12b**, *k*_obs_ = 8.8 × 10^−3^ s^−1^; mono-Al *k*_obs_ = 0.8 × 10^−3^ s^−1^; toluene, 120 °C, 1:50:100 catalyst:propylene oxide (PO):rac-LA. The mono-Zn and mono-Mg analogues were completely inactive under the same conditions, while Ca/Al and Li/Al complexes **12c-d** displayed lower activities than the mono-Al complex. Based on kinetic and computational studies (including ab initio molecular dynamics calculations), the high activity of **12a-b** was attributed to close intermetallic proximity, increased ligand strain and the rigid square pyramidal geometry around the Al centre (highest with **12b**), leading to improved monomer coordination. In addition, the Lewis acidity of Mg and Zn led to bridging Mg- or Zn-Cl-Al moieties thus longer and weaker Al–Cl bonds in **12a-b** (vs. mono-Al), which correlated with faster initiation (induction periods were 1, 3 and 4 min for **12a**,**b** and mono-Al, respectively).

### Transition metal/main group heterocombinations

**Fig. 3:**
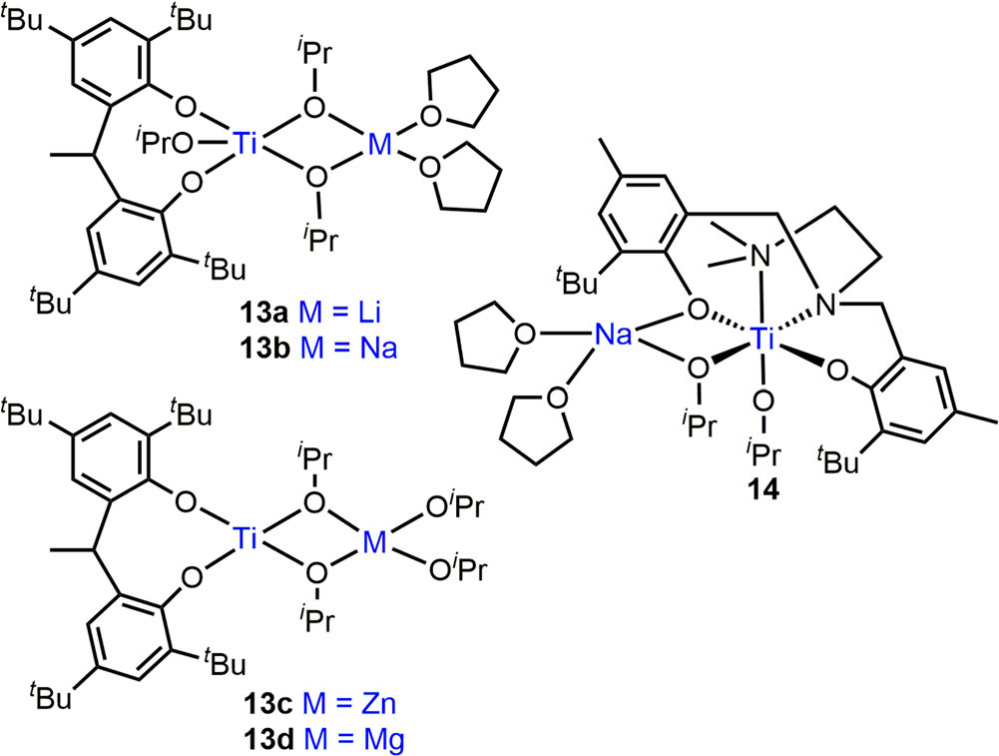
Heterometallic main group/Ti ROP catalysts. Structural representations of cyclic ester ROP catalysts combining Ti with alkali metals or divalent metals.

Main group/transition metal heterometallic ROP catalysts have so far focused on titanium, an attractive, non-toxic, inexpensive and abundant metal^47,48^. The development of heterometallic zirconium and hafnium catalysts offers an interesting area for future development, as both metals have excellent precedent in ROP^49,50^. The M/Ti(IV) complexes **13a-d** (M = Li, Na, Zn, Mg; Fig. 3) are efficient initiators for l-LA ROP in toluene at 30 °C^47^. The alkali metal-containing **13a-b** exhibited similar activity to the mono-Ti(IV) complex (76 equiv. l-LA converted in 94 h at 30 °C), despite X-ray crystallography of **13a** indicating increased Ti Lewis acidity, with longer and weaker (more nucleophilic) Ti-(O^*i*^Pr)_2_ bonds. Significant rate enhancements were however observed with **13c-d**, with **13c** (Zn/Ti) polymerising 91 equiv. l-LA within 30 min and **13d** (Mg/Ti) converting 89 equiv. l-LA in 3.5 h with good control (*Đ* = 1.27 (**13c**), 1.28 (**13d**)). The higher activity of **13c** vs. **13d** was originally attributed to the lower charge density of Zn than Mg resulting in weaker Zn-OR bonds than Mg-OR. However, due to the similarity in the ionic radii and charge (Zn^2+^ = 74 pm, Mg^2+^ = 72 pm)^51^, other factors such as the higher Lewis acidity and oxophilicity of Mg (*Ɵ* = 0.6) vs. Zn (*Ɵ* = 0.2) are likely to be key factors in explaining the metal-alkoxide bond strengths^52^.

Complex **14** bears a tetradentate ligand framework, and incorporation of Na was shown to increase the Ti Lewis acidity and weaken the Ti-O^*i*^Pr bonds (Fig. 3)^48^. Whilst only trace PCL was formed with the mono-Ti(III) and Ti(IV) analogues, **14** converted 182 equiv. ε-CL in 1 h (toluene, 60 °C), albeit with low polymerisation control (*Đ* = 2.5).

### F-block metal-based heterometallic complexes

ROP catalysts featuring f-block metals have also been developed to take advantage of their oxophilicity, Lewis acidity and large coordination spheres. Evidencing the role of lanthanide (Ln) metals in monomer activation, the activity of Na/Ln clusters ([Ln_2_Na_8_(OCH_2_CF_3_)_14_(THF)_6_]_,_ Ln = Sm, Y, Yb; **15a-c**, respectively)^53^ in ROP directly reflects the Ln ionic radius: **15a** > **15b** ≈ **15c** for ε-CL and **15a** > **15b** > **15c** for trimethylene carbonate (TMC) ROP. Notably, **15a** converted 3840 equiv. ε-CL in 30 min whereas only trace PCL formed with **15b-c** at [ε-CL]:[catalyst] loading of 4000:1. **15a** showed extraordinary activity for TMC ROP, converting 4000 equiv. in 1 min at 25 °C (*Đ* = 1.44). Heterometallic **15a-c** exhibited higher activity than the homometallic Ln phenoxide clusters and Na(OCH_2_CF_3_)^54^. The enhanced activity of **15a-c** was attributed to Na/Ln cooperativity via concurrent monomer activation and rapid ligand exchange. Moderate polymerisation control (*Đ* = 1.4-1.7) and shorter than expected *M*_n_ values were linked to initiation via multiple Ln-OCH_2_CF_3_ bonds and transesterification. **15a-c** were more active in toluene than in THF, which may suggest modification of the cluster structure in THF.

**Fig. 4:**
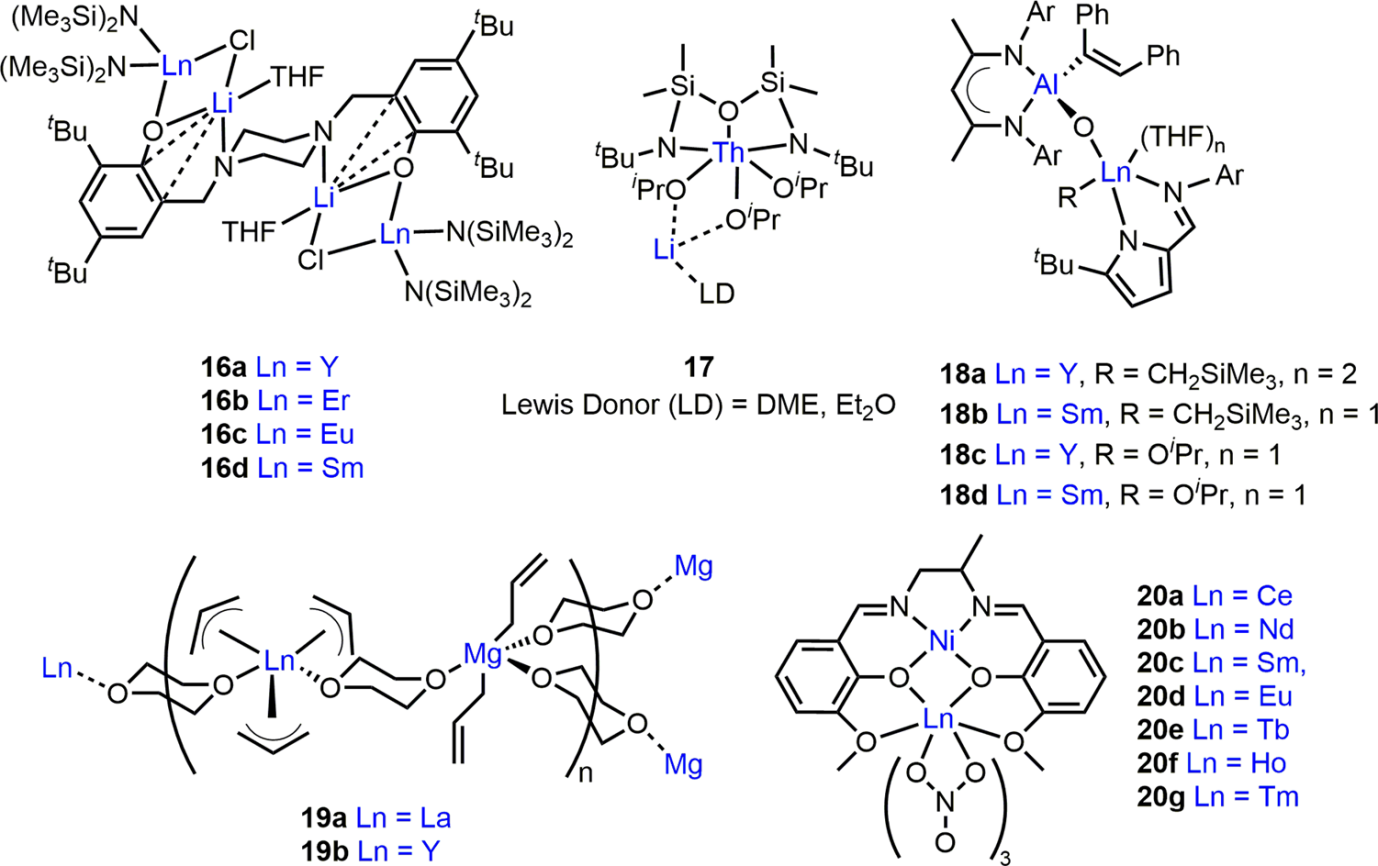
Heterometallic ROP catalysts featuring f-block elements. Structural representations of cyclic ester ROP catalysts combining lanthanides or actinides with alkali metals, divalent metals, trivalent metals or transition metals.

Similar trends were observed with Li_2_Ln_2_ (Ln = Y, Er, Eu and Sm) complexes **16a-d** (Fig. 4)^55^, with an activity decrease in l-LA ROP with decreasing Ln radius (**16d** > **16c** > **16b** ≈ **16a**). Complexes **16a** and **16d** were more active than the mono-Y and Sm analogues, which was tentatively attributed to reduced steric hindrance around Ln in **16a,d**. NMR analysis also suggested **16a** is more flexible than mono-Y, with the piperazidine ring in a chair conformation rather than a boat conformation. Similarly to clusters **15a-c**, **16a-d** showed higher activity and control in toluene than THF, which may indicate structural differences in Lewis donor solvents. Indeed, **16c** generated 98% PLA in toluene and 83% PLA in THF (30 min, 60 °C, [LA]:[Ln] = 1000:2), producing PLA with shorter than expected *M*_n_ in THF. It is plausible that **16a-d** dissociate in coordinating solvents to generate multiple initiating species, and/or that THF coordination to Li/Ln may inhibit monomer coordination^56^. The Li/Th(IV) complex **17** (Fig. 4) is the only actinide-based heterometallic ROP catalyst reported to date^57^. It displayed relatively low activity and polymerisation control, converting 48 equiv. l-LA in 2 h (toluene, 30 °C) with a bimodal *M*_n_ dispersity (*Đ* = 1.63).

Ln-based heterometallic ROP complexes have also been extended beyond alkali metals to Al^58^, Mg^59^ and Ni^60^. Heterometallic Ln/Al (Ln = Y, Sm) complexes **18a-d** (Fig. 4) were studied in *rac*-LA ROP^58^. While **18a** showed activity and control enhancements compared to the mono-Al and Y counterparts, converting 123 equiv. *rac*-LA in 5 h at 20 °C in toluene (*Đ* = 1.95), the reactivity of **18a** was further improved by addition of 5 equiv. hexamethyldisilazane (as a chain-transfer agent) or by alcoholysis with ^*i*^PrOH. The alkoxide analogues **18c-d** were highly active and both converted 495 equiv. *rac*-LA in 30 min with moderate control (*Đ* = 2.32 and 1.70, respectively). Strikingly, **18d** polymerised 1820 equiv. *rac*-LA in 1 h at 20 °C in toluene (*Đ* = 1.72), generating high *M*_n_ PLA (69 100 g/mol). The Al centre in **18a-d** was proposed not to be directly involved in ROP due to the low activity of mono-Al species (vs. **18c-d**), however Al may modulate the activity of Ln through electronic communication via the bridging O atom.

The activity of Ln/Mg allyl complexes **19a-b** (Ln = La or Y, respectively, Fig. 4) was explored in ε-CL and *rac*-LA ROP^59^. **19a** outperformed **19b**, converting 198 equiv. ε-CL in 20 s at 20 °C and 85 equiv. *rac*-LA in 2 h at 40 °C with good control (*Đ* < 1.4). While **19b** converted 122 equiv. ε-CL in 1.3 min at 20 °C, it was inactive for *rac*-LA ROP. The lower activity of **19b** was attributed to the smaller size of Y^3+^ (90 pm, vs. La^3+^_,_ 103 pm). Both complexes initiated ROP via nucleophilic attack of the allyl moiety on the coordinated monomer, as evidenced by ^1^H NMR spectroscopy.

Heterometallic Ln/Ni(II) complexes **20a-g** (Fig. 4, Ln = Ce, Nd, Sm, Eu, Tb, Ho and Tm) were tested for l-LA ROP^60^, however despite the improved control, all were less active than mono-Ni species. This was attributed to the Ln(NO_3_)_3_ moiety occupying the outer O_2_O_2_ salen cavity thus sterically hindering the monomer approach to Ni in the N_2_O_2_ core. Indeed, **20b** displayed the longest Ln-Ni distance (3.48 Å) and showed the highest activity, converting 730 equiv. l-LA in 24 h at 130 °C (*Đ* = 1.12).

### Key activity trends in ROP

Across heterometallic ROP studies, enhanced activity with larger and more Lewis acidic metals featuring more open coordination geometries emerges as one of the most prevalent trends. The highest activities are generally observed with medium/large metals, e.g. alkali metals and lanthanides, attributed to larger coordination spheres enhancing monomer coordination and activation. Combining Lewis acidic metals with more electronegative metals with weaker M-OR bonds may accelerate coordination and nucleophilic attack. Heterometallic complexes based on Al/Zn^46^, K/Zn^31^, La/Mg^59^, Li/In^38^, Li/Mg and Li/Zn^32^, Li/Sm^55^, Mg/Al^40^, Na/Sm^53^, Na/Zn^31^, Sm/Al^58^ and Ti/Zn^47^ have all displayed superior activities compared to the homometallic analogues. Most of these complexes feature a M-O-M′ framework, enabling intermetallic electronic communication and/or “ate”-type activation (vide supra), which can lead to enhanced nucleophilicity of the M-*R* bond (e.g. R = alkoxide)^38,47,48,58^. Despite these promising results, future ROP studies should explore the solution-state structure of heterometallic catalysts to confirm that the enhanced activities can be accredited to heterometallic cooperativity. Heterometallic catalysts should also be benchmarked against all homometallic counterparts to fully understand when heterometallic cooperativity leads to an activity enhancement.

While ROP provides a convenient route to access aliphatic polyesters and polycarbonates, these materials can also be accessed via epoxide ROCOP with cyclic anhydrides or CO_2_, respectively. ROCOP provides access to a broader scope of material properties due to the greater monomer structural diversity, and there is a growing interest in heterometallic ROCOP catalyst design. Owing to the mechanistic similarities between ROP and ROCOP, both of which include monomer coordination, nucleophilic attack and ring-opening, catalysts employed for these processes are often structurally-alike; some catalyst systems have demonstrated impressive activities in both^56,61–66^. Advances in heterometallic ROP catalysis may therefore inform future understanding and design of heterometallic ROCOP catalysts and vice versa.

### Ring-opening copolymerisation

Epoxide ROCOP with CO_2_ or cyclic anhydrides displays sustainability benefits, as CO_2_ may be sourced from industrial waste streams^16,67,68^, and some epoxides and anhydrides can be derived from biomass (e.g. limonene/α-pinene oxide, succinic/citraconic anhydrides)^69,70^. Life cycle analysis has suggested that incorporating CO_2_ into polyol production (for subsequent polyurethane synthesis) can reduce petrochemical consumption by 20% and CO_2_ emissions by 19% compared to conventional polyol synthesis^71^.

Catalyst design has enabled the generation of nearly perfect polycarbonates (>99% carbonate linkages) through ROCOP, by overcoming the undesired formation of polyether linkages and cyclic carbonates^70,72^. Most catalyst systems reported are homometallic and often multimetallic^64,70,73^, including bis-Co(II)^74^, Co(III)^75,76^, Fe(III)^77^, Mg(II)^67^ and Zn(II) complexes^78–83^. Studies have pointed towards a bimetallic mechanism, which limits the use of dimeric catalysts with high monomer loadings or at high dilutions. Intermetallic proximity in the range of 3–5 Å is generally optimal for improved catalyst performance, which has directed ligand design towards dinucleating scaffolds^77,84^. Mechanistic studies on homobimetallic complexes suggest that both metals are involved in ROCOP, and hint that heterometallic catalysts could further improve activities by enhancing both epoxide coordination (Lewis acidic/electrophilic metals) and the nucleophilicity of the metal-carbonate bond. Examples of heterobimetallic catalysts that combine main group, transition and Ln metals have recently been reported.

### Main group heterometallic complexes in epoxide/CO_2_ ROCOP

**Fig. 5:**
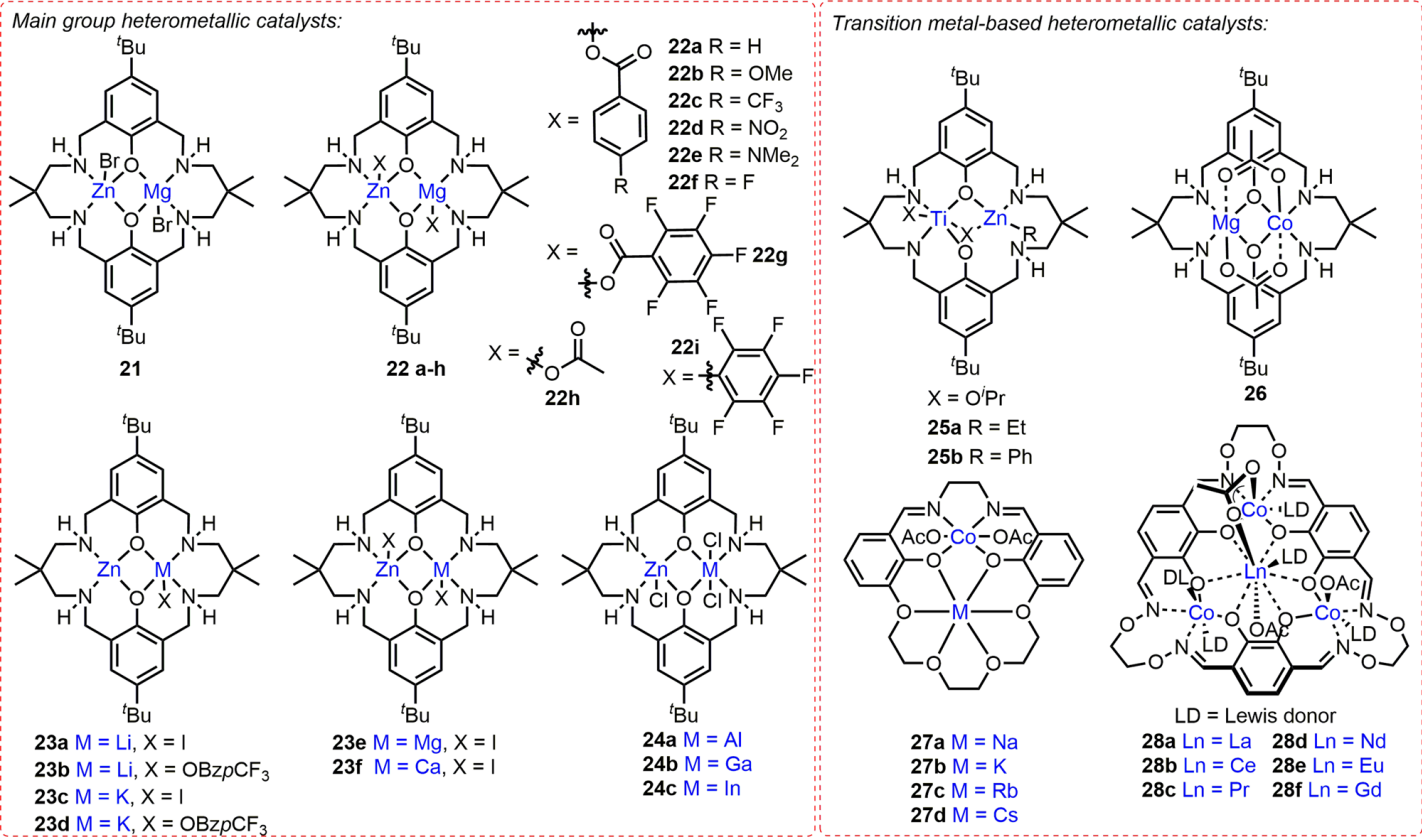
Main group and transition metal heterometallic complexes for epoxide ROCOP with CO_2_ or cyclic anhydrides. Structural representations of ROCOP catalysts that feature heterometal combinations from across the s, p and d-block.

Initial studies on homogeneous heterometallic catalyst systems investigated a mixture of **L**Zn_2_(OAc)_2_:**L**ZnMg(OAc)_2_:**L**Mg_2_(OAc)_2_ in an assumed 1:2:1 ratio (**L** = macrocyclic diphenolate tetramine ligand of **21-26**, Fig. 5). This mixed system was twice as active (TOF = 79 h^−1^) as a 1:1 mixture of **L**Zn_2_(OAc)_2_:**L**Mg_2_(OAc)_2_ in CHO/CO_2_ ROCOP (0.1 mol% catalyst loading, neat epoxide, 1 bar CO_2_, 80 °C)^85^. The first pure homogeneous heterometallic ROCOP catalyst reported was **L**ZnMgBr_2_ (**21**, Fig. 5)^86^, which displayed TOF = 34 h^−1^ in CHO/CO_2_ ROCOP. **21** was twice as active as **L**Mg_2_Br_2_ and five times faster than the 1:1 **L**Mg_2_Br_2_:**L**Zn_2_Br_2_ mixture, whereas **L**Zn_2_Br_2_ was inactive (0.1 mol% catalyst loading, neat epoxide, 1 bar CO_2_, 80 °C). **21** displayed relatively good polymerisation control, generating >99% carbonate linkages with only trace cyclic carbonate, albeit with a bimodal dispersity (*Đ* = 1.14), which is common in the field and is often attributed to the presence of diol impurities acting as chain-transfer agents to produce telechelic polymers^87^.

**Fig. 6:**
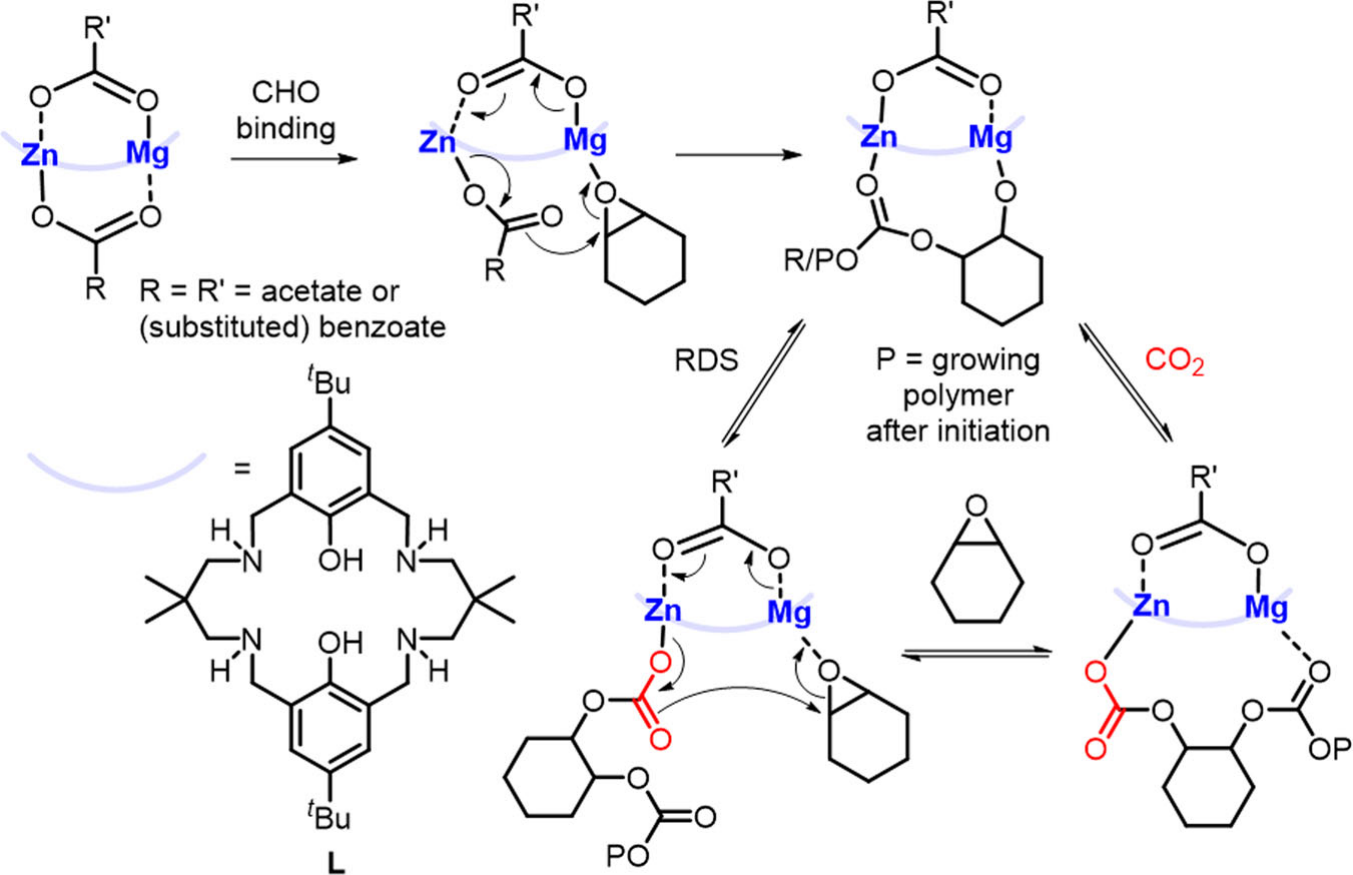
Heterometallic chain-shuttling mechanism. Proposed mechanism for CHO/CO_2_ ROCOP with heterometallic complexes **22a-h**^88^.

Metathesis of **21** with potassium carboxylates generated Mg/Zn complexes **22a-h** with acetate/benzoate co-ligands (Fig. 5)^88^. Kinetic studies showed that switching the co-ligand from Br to *para*-NO_2_ benzoate reduced the induction period from 160 min (**21**) to 20 min (**22d**) and enhanced the propagation rate from 3.0 × 10^−5^ s^−1^ to 3.8 × 10^−5^ s^−1^, respectively. **22a-h** displayed excellent selectivities (>99% carbonate linkages) and retained their heterometallic structure in THF-*d*_8_, suggesting that the structures are likely to be retained during polymerisation (neat epoxide). The high activities of **21** and **22a-h** were attributed to cooperative Mg/Zn catalysis via a chain-shuttling mechanism (Fig. 6), with Lewis acidic Mg enhancing epoxide coordination and the labile Zn-carbonate bond accelerating the nucleophilic attack. Recently, **22i** catalysed the one-pot RO(CO)P of bio-based ε-decalactone with CHO/CO_2_ in the presence of 4 equiv. 1,2-cyclohexane diol, giving >90% monomer conversions^89^. The resultant degradable poly(cyclohexene carbonate-*b*-decalactone-*b*-cyclohexene carbonate) terpolymers displayed >99% CO_2_ selectivity and molar masses ranging from 38–71 kg/mol (*Đ* < 1.16), with improved material properties compared to poly(cyclohexene carbonate).

**L**MZnX_2_ complexes **23a-f**^90^ and **24a-c**^91^, combining Zn with s- and p-block heterometals, were also tested in ROCOP (Fig. 5). With **23e-f**, epoxide activation on Mg or Ca (respectively) was proposed as X-ray crystallographic studies showed THF coordination to these metals. Heterometallic **23e** (Mg/Zn) displayed a TOF of 72 h^−1^ and was six times more active than **L**Zn_2_I_2_ (0.1 mol% catalyst loading, neat epoxide, 1 bar CO_2_, 80 °C). However, all other heterocombinations investigated were less active than **L**Zn_2_I_2_. As well as the heterometal selected, the ligand conformation and the intermetallic separation may also play a role, as these differed with Li (**23b**, “crown-shape”; Zn–Li = 2.86 Å), Mg (**23e**, “bowl-shape”; Zn–Mg = 3.06 Å) and Ca (**23** **f**, “S-shaped”; Zn–Ca = 3.35 Å). Interestingly, only **23b**,**d**-**e** were selective for polycarbonate formation (>96%), with **23a**,**c**,**f** generating *cis*-cyclic carbonates (>99%). Cyclic carbonate formation was linked to the iodide lability and potential dissociation of the growing polymer chain from the metal due to the increased ionic character of the metal carbonate bond^92^.

The importance of the metal combination in CHO/CO_2_ ROCOP was also evident with complexes **24a-c**^91^, where **L** adopts a “bowl” conformation with three chloride co-ligands. Heterometallic **24a-c** were less active and selective than **L**Zn_2_Cl_2_ (TOF = 9 h^−1^, 0.1 mol% catalyst loading, neat epoxide, 1 bar CO_2_, 80 °C), with significant polyether formation (up to 32%). Increasing the CO_2_ pressure to 20 bar led to a perfectly alternating polycarbonate with **24c**, suggesting that CO_2_ insertion may be implicated in the rate-limiting step, unlike **L**-based bis-Zn and Mg/Zn complexes, which are generally zero-order in CO_2_^82,88^. The CO_2_ uptake (Al < Ga < In) increased with the decrease in the Lewis acidity of the Group 13 metal^91^. This observation, along with the increased lability of the metal-alkoxide intermediates, was used to explain the activity increase from Al (TOF = 1 h^−1^) to In (TOF = 9 h^−1^). These features also outweighed the influence of intermetallic proximity on the activity of **24a-c**, with both the distance and activity increasing on descending Group 13 (Zn–Al = 3.02 Å, Zn–Ga = 3.12 Å, Zn–In = 3.15 Å).

### Main group and transition metal heterometallic complexes in epoxide/CO_2_ ROCOP

Heterometallic main group/transition metal complexes based on **L** have also been synthesised (**25a-b**^63^ and **26**^93^, Fig. 5). Both **25a-b** (Ti/Zn) showed low to moderate activity, selectivity and polymerisation control in CHO/CO_2_ ROCOP with TOF = 3 h^−1^, ~94% carbonate linkages and bimodal dispersity (*Đ* = 1.35 and 1.37, respectively; 1 mol% catalyst loading, 1 bar CO_2_, 80 °C)^63^. Notably, the analogous mono-Ti and mono-Zn complexes were inactive in CHO/CO_2_ ROCOP, highlighting the benefit of heterometallic Ti/Zn^86^. **25a-b** were also moderately active in ROP, converting up to 89 equiv. l-LA in 40 min (*Đ* = 1.13) and 94 equiv. ε-CL in 90 min (*Đ* = 1.21-1.33) at 70 °C in THF^63^.

The Mg/Co complex **26** (Fig. 5) recently outperformed all **L**-supported heterometallic complexes in CHO/CO_2_ ROCOP, with TOF of 455 h^−1^ at 1 bar CO_2_ pressure (0.1 mol% catalyst loading, neat epoxide, 80 °C), > 99% carbonate linkages and good polymerisation control (*Đ* = 1.13)^93^. Under identical conditions, **26** was almost five times more active than **22** **h** (Mg/Zn), suggesting that Co is more active than Zn in ROCOP. **26** was also twice as active as **L**Co_2_(OAc)_2_, and three times faster than **L**Mg_2_(OAc)_2_ (0.1 mol% catalyst loading, neat epoxide, 1 bar CO_2_, 120 °C). These observations were supported by kinetic studies, as the transition state Gibbs free energy (ΔG‡) and enthalpy barriers (ΔH‡) were lowest for **26** (ΔG‡ = 94.5 ± 1.2 kJ mol^−1^, ΔH‡ = 77.3 ± 1.2 kJ mol^−1^) and highest for **L**Mg_2_(OAc)_2_ (ΔG‡ = 100.2 ± 1.3 kJ mol^−1^, ΔH‡ = 83.3 ± 1.3 kJ mol^−1^), implying that Co accelerates nucleophilic attack by lowering the ΔH‡. Conversely, the entropy ΔS‡ values were reduced for **26** and **L**Mg_2_(OAc)_2_ (ΔS‡ = −46.1 ± 3.4 J mol^−1^ and −45.4 ± 3.7 J mol^−1^, respectively) vs. **L**Co_2_(OAc)_2_ (ΔS = −60.2 ± 4.2 J mol^−1^)^93^. This was attributed to the lower bond directionality of Mg, possibly resulting in increased degrees of freedom during epoxide coordination. **26** was therefore proposed to catalyse ROCOP via a chain-shuttling mechanism (Fig. 6) with CHO coordination to Mg, followed by ring-opening via the Co-carbonate bond.

The M/Co complexes **27a-d** (M = Na, K, Rb or Cs, respectively) were recently reported as the first heterometallic catalysts to exhibit good turnover numbers for PO/CO_2_ ROCOP^94^. **27b** (K/Co) was most active, displaying TOF of 340 h^−1^ vs. **27a** (TOF = 120 h^−1^), **27c** (TOF = 54 h^−1^) and **27d** (TOF = 47 h^−1^) in neat PO (0.025 mol% catalyst loading, 50 °C, 20 bar CO_2_ pressure). Monomodal SEC traces were obtained in the presence of >50 equiv. of 1,2-cyclohexane diol as a chain-transfer agent, with controllable *M*_n_ values between 1.3 and 79.6 kg/mol. **27b** also displayed an outstanding TOF of 800 h^−1^ (0.025 mol%, 70 °C, 30 bar CO_2_ pressure), with narrow dispersity (*Đ* = 1.07), >99% CO_2_ uptake and 93% polycarbonate selectivity. The highest activity of **27b** was attributed to the optimal combination of metal sizes and binding affinities. While smaller Na may be coordinatively saturated by the crown ether, preventing PO coordination, the larger Rb and Cs in **27c-d** prevent coplanar incorporation into the macrocycle and form aggregate structures. From the kinetic data, the rate-determining step was proposed to involve ring-opening of K-coordinated PO by a Co(III)-carbonate intermediate via a chain-shuttling mechanism akin to other heterometallic catalysts (Fig. 6). Notably, **27b** was also more than twice as active as Mg/Co complex **26** in CHO/CO_2_ ROCOP (*k*_p_ = 31.7 mM^−1^ s^−1^ at 50 °C for **27b** and *k*_p_ = 15.1 mM^−1^ s^−1^ for **26** at 60 °C).

The Ln/Co_3_ complexes **28a-f** (Ln = La, Ce, Pr, Nd, Eu or Gd, respectively, Fig. 5) were recently studied in CHO/CO_2_ ROCOP^95^. Both Ln and Co were proposed to act as Lewis acids, based on MeOH and H_2_O coordination in the solid-state structures. **28a-f** significantly outperformed their monometallic counterparts (monometallic Co(II) complex and La(OAc)_3_), which yielded only trace polycarbonate, either alone or in combination, whereas **28a** displayed a TOF of 1375 h^−1^ with >99% carbonate linkages and narrow (bimodal) dispersity (*Đ* = 1.04/1.04; 8.0 × 10^−4^ mmol% catalyst loading, neat epoxide, 20 bar CO_2_, 130 °C). **28d** with medium-sized Nd displayed the highest activity (TOF = 1625 h^−1^; *Đ* = 1.05/1.04). **28a-f** were proposed to promote ROCOP by CHO coordination to the oxophilic Ln, followed by epoxide ring-opening by the Co-acetate/-carbonate bond. The resulting Ln-alkoxide was then proposed to form carbonate species with Co-bound CO_2_, leading to chain propagation.

### Heterometallic complexes containing lanthanide elements in epoxide/CO_2_ ROCOP

**Fig. 7:**
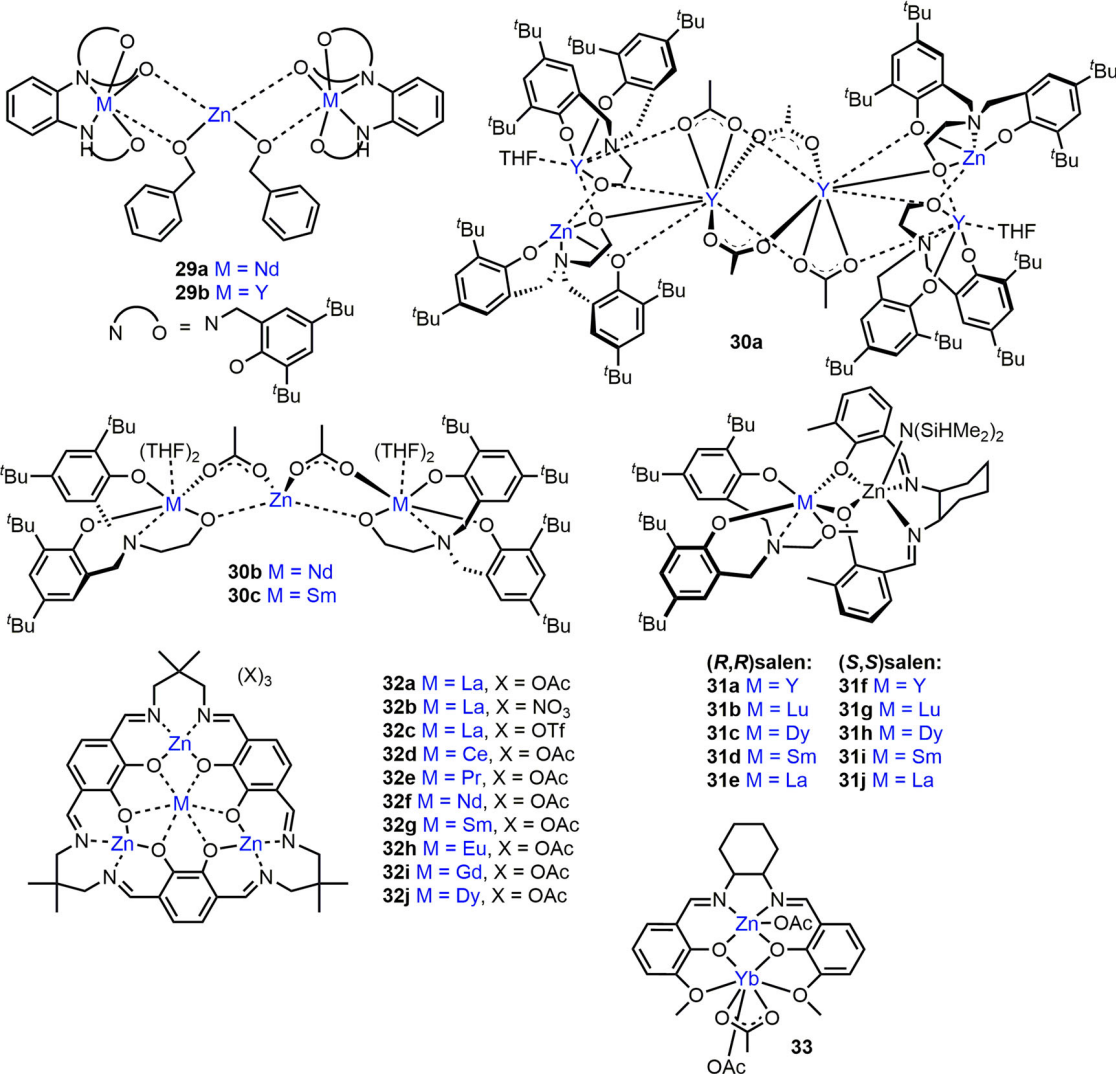
Heterometallic Ln/Zn complexes for epoxide ROCOP with CO_2_ or cyclic anhydrides. Structural representation of zinc-based ROCOP catalysts featuring lanthanide heterometals from across the f-block.

Epoxide activation is a key ROCOP step, and Ln metals have displayed good monomer coordination in ROP^53,55,58,59^. A range of Ln/Zn complexes have also been developed for CHO/CO_2_ ROCOP, including complexes **29a-b** (Ln = Nd, Y)^96^, **30b-c** (Ln = Y, Nd or Sm)^97^ and **31a-j** (Ln = Y, Lu, Dy, Sm or La)^98^ (Fig. 7). **29a** (Nd_2_/Zn) showed good activity with maximum TOF = 273 h^−1^ (ref. ^96^), 99% carbonate linkages and moderate polymerisation control (*Đ* = 1.81; 0.1 mol% catalyst loading, [epoxide] = 4.92 M in toluene, 7 bar CO_2_, 70 °C). Conversely, **29b** (Y_2_/Zn) was significantly slower and less selective with TOF of 33 h^−1^ and 63% carbonate linkages, likely linked to the smaller size of Y^3+^ (90 pm) vs. Nd^3+^ (99 pm) giving poorer epoxide coordination. While ROCOP catalysts typically require elevated temperatures, **29a** also displayed good activity at ambient temperature with TOF = 82 h^−1^ (*Đ* = 1.65) under otherwise identical conditions. The analogous mono-Nd complex and ZnEt_2_/BnOH mixture failed to initiate ROCOP (7 bar CO_2_, 70 °C), highlighting the cooperativity of the Nd/Zn system.

The Ln/Zn complexes **30a-c** (Fig. 7, Ln = Y, Nd or Sm, respectively) displayed moderate activity and selectivity in CHO/CO_2_ ROCOP^97^. The most active complex, **30a**, required 24 h to generate 71% polycarbonate (toluene, 30 bar CO_2_, 70 °C) but outperformed the homometallic Y complex, which showed negligible activity. High CO_2_ pressures were required for polycarbonate synthesis; only 16% polycarbonate was formed with **30a** at ambient pressure, and the poor polymerisation control of **30a-c** (*Đ* = 8.42–9.50) was attributed to polymer degradation and cyclic carbonate/polyether formation.

The Ln ionic radii in zincate complexes **31a-j** (Fig. 7) significantly influenced their activity in CHO/CO_2_ ROCOP^99^; **31c-d**,**h-i** featuring medium-sized Dy and Sm were most active (TOF = 124 h^−1^), generating perfectly alternating polycarbonates with moderate control (*Đ* = 1.52–1.62, 1500:1 [CHO]:[catalyst], 30 bar CO_2_, 70 °C). Structural analysis of **31d** indicated close Sm-Zn proximity (3.47 Å) and elongated Zn-phenoxide bonds, suggesting **31d** might be more nucleophilic than both mono-Sm and mono-Zn complexes. Indeed, the homometallic counterparts of **31c-d**,**h-i** showed low or no activity under the same conditions. Low to moderate activities were observed with **31a-b**,**f-g** (larger Ln) and no polymer was formed with **31e,j** (smallest Ln).

Studies of Ln/Zn complexes **32a-j** (Ln = La, Ce, Pr, Nd, Sm, Eu, Gd, Dy, Fig. 7) in CHO/CO_2_ ROCOP highlight the importance of the anionic co-ligand, as well as the Ln size^98^. While acetate complex **32a** exhibited TOF = 230 h^−1^ and generated polymers with >99% carbonate linkages, the nitrate analogue **32b** formed trace polymer and trifluoroacetate complex **32c** favoured polyether formation (neat epoxide, 10 bar CO_2_, 100 °C). The higher activity of **32a** was attributed to the rapid exchange of the coordinated and outer-sphere acetate anions. The highest catalyst activities and selectivities were observed with **32a-f**, which featured larger Ln metal centres than **32g-j**. **32d** (Ce/Zn) showed the highest activity with TOF = 370 h^−1^ (*Đ* = 1.3).

All Ln/Zn complexes reported for CHO/CO_2_ ROCOP were proposed to operate via a chain-shuttling mechanism (Fig. 6), with the Lewis acidic Ln metal activating CHO for nucleophilic attack by the labile Zn-carbonate, formed via CO_2_ insertion into the Zn-alkoxide bonds. Due to mechanistic similarities, it is plausible that the heterometallic cooperativity of catalysts reported for epoxide/CO_2_ ROCOP could be extended to epoxide/cyclic anhydride ROCOP, to form a series of polyesters by switching the carbonyl source^70^.

### Heterometallic complexes in epoxide/cyclic anhydride ROCOP

To date only two heterometallic complexes, **21**^86^ (Mg/Zn, Fig. 5) and **33**^100^ (Yb/Zn, Fig. 7), have been explored in epoxide/anhydride ROCOP. **21** displayed promising activity in CHO/phthalic anhydride ROCOP, likely operating via a similar chain-shuttling mechanism proposed for CHO/CO_2_ ROCOP (Fig. 6), with TOF = 188 h^−1^ (ref. ^86^), which was 40 times higher than the 1:1 **L**Mg_2_Br_2_:**L**Zn_2_Br_2_ mixture (1 mol% catalyst loading, neat epoxide, 100 °C). **21** also showed excellent selectivity for polyester formation (>99%) and good polymerisation control with *M*_n_ values up to 10 900 g/mol (bimodal *Đ* = 1.04, 1.09). **33** was tested in CHO/maleic anhydride (MA) ROCOP with and without a co-catalyst (4-dimethylaminopyridine (DMAP) or triphenylphosphine (TPP))^100^. In the absence of a co-catalyst, **33** converted 17% CHO in 2.5 h at 110 °C (250:250:1 CHO:MA:**33**), giving high polyether content (61%). With TPP, **33** converted 73% CHO in 6 h at 110 °C, yielding a perfectly alternating polyester with *M*_n_ up to 12 830 g/mol (*Đ* = 1.13; 250:250:1:1 CHO:MA:**33**:TPP). Using the more nucleophilic DMAP co-catalyst, **33** converted 90% CHO under the same conditions but gave lower polyester selectivity (31% polyether linkages).

### Aziridine/CO ROCOP

**Fig. 8:**
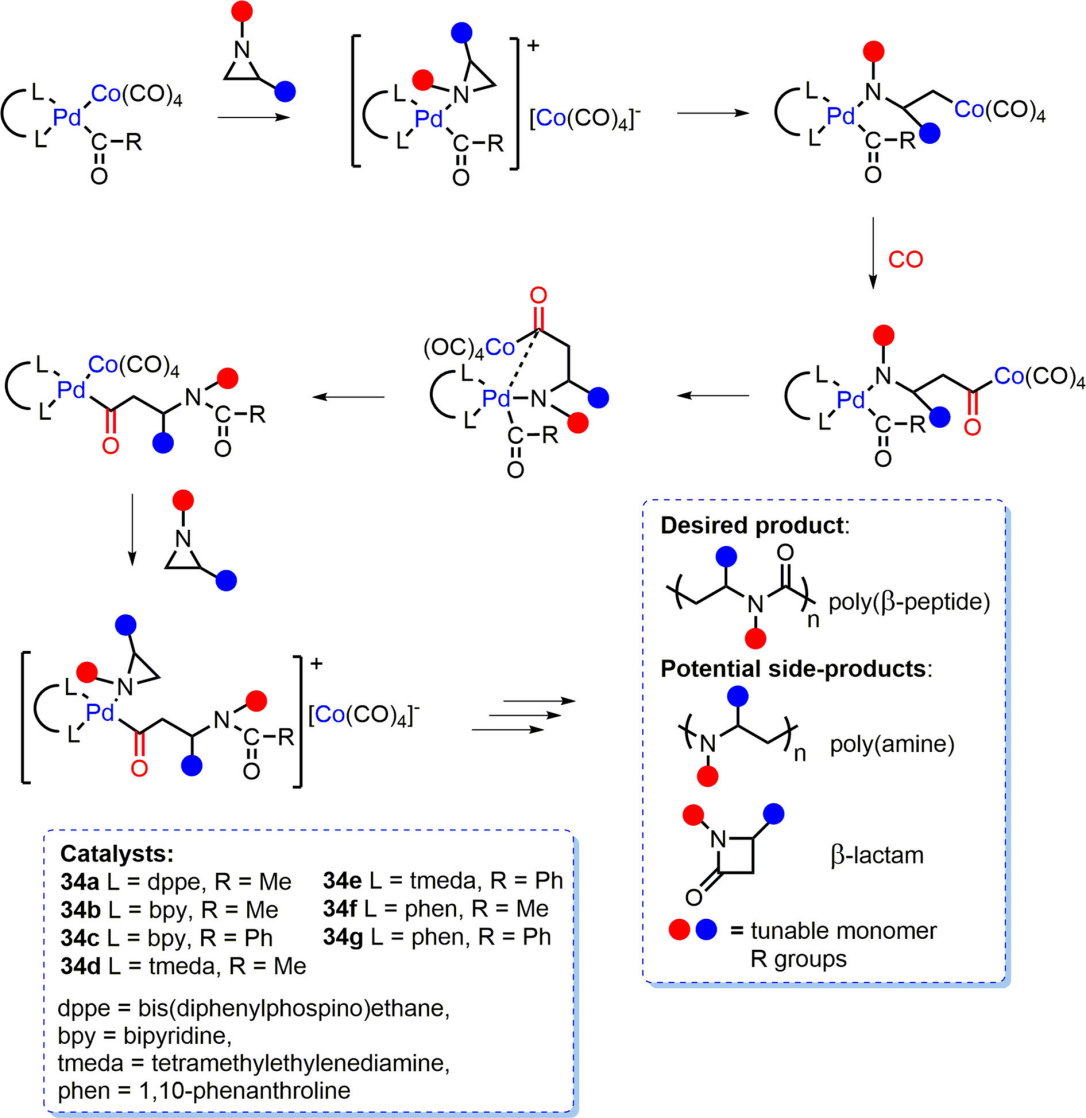
Proposed aziridine/CO ROCOP mechanism for Co/Pd catalyst systems. Proposed heterometallic mechanism for complexes **34a-g** along with targeted poly(β-peptide) product and potential side-products from aziridine/CO ROCOP^109^.

Aziridine/CO ROCOP is an underexplored route towards poly-β-peptides and polypeptoids (*N*-alkylated polymer) with potential applications in catalysis, materials and biomedicine^101–104^. Suitable catalysis is required to produce poly-β-peptides/polypeptoids and to overcome the formation of side-products such as β-lactams and polyamines (Fig. 8)^105–107^. Mono-Co systems have been most explored but have generally displayed poor polymerisation control (*Đ* ≈ 11.5)^108^ and low selectivity for alternating aziridine/CO enchainment, requiring high CO pressures (<69 bar)^105^.

Co/Pd complexes **34a-g** (Fig. 8, Co-Pd distances ≈ 2.6 Å) are the only heterometallic catalysts reported for aziridine/CO ROCOP^109^. **34a** exhibited the highest activity for both substituted and non-substituted aziridines, yielding 69% copolymer from 2-methylaziridine in 6 h at 100 °C and 50 bar CO pressure (*Đ* = 1.5; 1 mol% catalyst). Unfortunately, the catalyst selectivity for copolymer production was not assessed. However, **34a-g** were significantly more active than monometallic [Co(C(=O)Me)(CO)_3_(PPh_3_)], [Co(C(=O)CH_2_Ph)(CO)_4_]^105,108^ and [PdMe(NCMe)(bpy)]^+^[BF_4_]^–^ complexes, which was attributed to Co/Pd cooperativity. **34a-g** were proposed to operate via reversible aziridine coordination to Pd, generating the [PdC(=O)R(aziridine)(L)]^+^[Co(CO)_4_]^–^ (R = Me, Ph; L = dppe, bpy, tmeda or phen) ion pair, followed by aziridine ring-opening via nucleophilic attack of Co^–^ upon the methylene carbon of the aziridine and subsequent CO insertion (Fig. 8).

### Key activity trends in ROCOP

The nature of the metal combination and the initiating nucleophile are key in epoxide/CO_2_, epoxide/anhydride and aziridine/CO ROCOP. Generally, the different metals are proposed to adopt distinct roles, with the more Lewis acidic metal(s) activating the monomer(s) and the more labile metal-oxygen bonds accelerating nucleophilic attack^63,86,88,90,91,93,95^. While many heterometallic complexes have led to enhanced activities, selectivities and polymerisation control, not all heterocombinations give improved performance, and a careful balance of Lewis acidity and M-OR bond polarity is required. Similarly to the trend observed in ROP, the catalytic activity of Ln-based heterometallic catalysts in ROCOP is often linked to Ln size; Ln with medium/large ionic radii (e.g. Ce and Sm) generally increase epoxide coordination and catalyst activity^96–98^. Notably, most heterometallic ROCOP catalysts operate without a co-catalyst, enabling the synthesis of high molar mass polymers, albeit often with the aforementioned bimodal dispersity.

### Summary and outlook

In both ROP and ROCOP, heterometallic catalysts have displayed reactivity enhancements by facilitating concurrent monomer activation and nucleophilic attack. However, not all heterometallic combinations improve catalyst performance. Whilst more studies are required to fully understand and predict cooperative heterocombinations, key RO(CO)P catalyst features have started to emerge, including the metal size and coordination number, M-OR bond strength, M–M′ proximity, and the solution-state catalyst structure under polymerisation conditions.

Pairing a hard metal (M, e.g. Group 1, 2 and Ln) with a softer, more carbophilic metal (M′, e.g. Co and Zn) can result in anionic “ate” activation, with the transfer of anionic ligands to the more carbophilic metal. For example, complexes **3a-b** and **4a-d** bear structures typically referred to as higher order zincates/magnesiates, where the central carbophilic Zn^2+^/Mg^2+^ is surrounded by four anionic ligands. While not all of the complexes discussed herein bear typical “ate” structures, almost all feature a bridging M-O-M′ unit that enables electronic communication between the two metal centres. This electronic communication has the potential to form complexes with at least a partial “ate” character, with the tug of war of electron density between M and M′ lying to the side of M′. This unequal sharing of the electron density may simultaneously increase the Lewis acidity of the M centre and also the nucleophilicity of the M′-*R* group (where R = e.g. an alkoxide); both are key catalyst features in ROP and ROCOP. The concept of heterometallic cooperativity has been relatively well-exploited with heterometallic complexes in epoxide/CO_2_ ROCOP, where the two metals typically adopt different roles of epoxide coordination and nucleophilic attack from a metal-carbonate group in the chain-shuttling mechanism. The larger Lewis acidic metal (e.g. K, Mg or Ln) usually enhances Lewis donor (e.g. monomer) coordination, as observed by the preferential coordination of THF to Mg in **23e** (Mg/Zn)^90^ and MeOH/H_2_O to the Ln centre in **28a-f** (Ln/Co)^95^ in the molecular structures. Studies with **26** (Mg/Co) and **27b** (K/Co) suggest that the more Lewis acidic Mg/K enhances the role of epoxide coordination, whereas the more electronegative metals (e.g. Co and Zn) typically accelerate the nucleophilic attack and epoxide ring-opening^93,94^, lowering the transition state barriers vs. the homometallic counterparts. Many of the reported heterometallic ROCOP catalysts feature heterometals in close proximity, typically within the 3–5 Å range proposed to be crucial for effective bimetallic electronic communication and the chain-shuttling ROCOP mechanism^77,84^. Catalysts designed for cyclic ester/cyclic carbonate ROP and CO_2_/epoxide ROCOP share several key features, including the Lewis acidity of the metal and nucleophilicity of the M–O(polymer) bond. Studies on bimetallic ROP catalysts suggest that intermetallic proximity can also be important, however further mechanistic investigations are required. While the individual heterometal roles are less well understood in ROP, the highest activities have been observed with complexes where medium/large Group 1/Ln metals are combined with Zn. Complexes comprising larger Lewis acidic Group 1/2/Ln metals have generally displayed superior activities, attributed to the presence of additional monomer coordination sites. Based on the mechanistic similarities between ROP and ROCOP, it is plausible that in the presence of heterometallic complexes, ROP also proceeds *via* Lewis acidic activation of the monomer by the Group 1/Ln metals, followed by nucleophilic attack and monomer insertion at the more electronegative M′-OR bond. This hypothesis is supported by structural analysis showing THF coordinated to alkali metals in **2**, **3a-b**, **4a-d**, **5a-b**, **6a-d**, **13a-b**, **14** and **16a-d** and to Ln metals in **18a-d**.

Heterometallic catalyst performance is also influenced by the reaction conditions; the solvent (or monomer) may alter the complex aggregation state or promote the in situ formation of solvent separated ion pairs. Throughout heterometallic RO(CO)P, there are examples where THF solvent decreases the catalyst activity, and further studies are required to understand whether this stems from increased M–M′ distances (by in situ modification of solution-state heterometallic structures) and/or competitive THF metal coordination blocking the monomer approach. Understanding whether heterometallic complexes maintain their structure in the solution-state is therefore crucial.

Alkali and Ln-based heterometallic catalysts have typically led to high activities in RO(CO)P, and there is scope for catalyst development with di-, tri- and tetravalent metal heterocombinations. Many of the heterometallic systems reported thus far have also been investigated under different reaction conditions, which makes comparisons challenging. This is especially prominent in ROP, and thus systematic studies are required to identify superior and inferior heterocombinations.

Heterometallic cooperativity has the potential to shape future RO(CO)P catalyst design. At present, heterometallic catalyst performance often falls short of the homometallic frontrunners, however there has been far more extensive research into homometallic catalysts, with >100 homobimetallic examples reported for LA ROP (vs. 39 heterometallic catalysts). While heterometallic catalyst synthesis is sometimes regarded as challenging, different methodologies including sequential deprotonation, coordination and/or transmetalation reactions, and reactions with preformed “ate” complexes have been successfully employed to prepare heterometallic complexes. Promisingly, we are now reaching a turning point, with a recently reported Mg/Co system now amongst the most active epoxide/CO_2_ ROCOP catalysts at atmospheric CO_2_ pressure. More recently, DFT calculations have been used to understand which catalyst features lead to heterometallic cooperativity in RO(CO)P, and there are exciting opportunities to use computational approaches to both understand and predict cooperative heterometallic catalysis in the future. The studies described here signal clear directions for understanding and exploiting heterometallic cooperativity within RO(CO)P catalysis.
